# Associations Between Aspects of Diet and Internalizing and Externalizing Behaviors in Children Aged 4 Years

**DOI:** 10.3390/nu18091461

**Published:** 2026-05-02

**Authors:** Nina Cecilie Øverby, Elisabet Rudjord Hillesund, Christine Helle

**Affiliations:** Department of Nutrition and Public Health, University of Agder, 4604 Kristiansand, Norway; nina.c.overby@uia.no (N.C.Ø.); elisabet.r.hillesund@uia.no (E.R.H.)

**Keywords:** child mental health, internalizing and externalizing behaviors, diet, fruit and vegetables, non-core foods, sugar-sweetened beverages

## Abstract

Background/objectives: Mental health challenges are increasing worldwide. Identifying preventable factors for such challenges is important and will have the greatest impact if identified in young children. In this study we aimed to explore the association between aspects of diet and child mental health at the age of 4 years. Methods: This is a secondary analysis of data from the Early Food for Future health study in Norway, a randomized controlled trial aiming to improve diet at ages 6–12 months. Cross-sectional data from 363 children aged 4 years and their mothers are used. Diet was assessed using food frequency questions. Five food scores were developed: vegetables, fruits, fruit and vegetables, sweet/salty snack score, and soft drink score. The Child Behavioral Checklist (CBCL) was used to assess internalizing and externalizing behaviors. Crude and multivariable linear regression models are presented. Results: We found that frequency of consumption of vegetables and total frequency of consumption of fruit and vegetables in 4-year-old children were inversely associated with internalizing behavior (β −0.207, 95% CI: 0.351, −0.063), while frequency of consumption of sweet and salty snacks was positively associated (β 1.807, 95% CI: 0.276, 3.337) with externalizing behavior and frequency of consumption of fruit and vegetables was inversely associated (β −0.188, 95% −0.336, −0.041). All were independent of maternal education, measures of financial difficulties and maternal mental health. Conclusion: An inverse association was observed between child frequency of consumption of fruit and vegetables and internalizing and externalizing behaviors, whereas frequency of consumption of sweet/salty snacks was positively associated with externalizing behavior. A varied and healthy diet early in life may promote child mental health, with potential large returns for society. Given the observational nature of the data, causal inference is limited and intervention studies are needed.

## 1. Introduction

There is a rising concern about population mental health worldwide, as numbers of people with adverse mental health are increasing [1]. Mental health is an essential part of total health and wellbeing [1]. Mental health is specifically important early in life as it is additionally critical for school outcomes, social skills and education attainment factors, which influence lifelong health and social and economic outcomes [2]. Today, one in seven young people are estimated to be living with mental health disorders [3], affecting both individuals, their families and society. Among young people, internalizing problems, defined as symptoms of depression, anxiety and emotional problems, are frequently reported [4]. Externalizing problems entail aggressive and oppositional behavior and predict social problems and poor academic achievement throughout childhood and adolescence [5]. In international mental health surveys, around 13% of youth have externalizing problems of clinical concern [2]. Around two thirds of children showing externalizing problems between ages 2 and 3 years continue to show high levels into school age; however, less than 10% start to have conduct problems after age 5 years [6]. This shows the importance of identifying factors relevant to mental health early in life.

Psychological and social factors are well known to be involved in child mental health. This includes parental mental health and practices which compromise caregiving quality [6]. In addition to this, recent research shows that food consumption and diet quality play an important role in mental disorders [7]. Typically, nutrients like iron, iodine and long-chain fatty acids have been central in the research field due to their individual and crucial role in brain development [8]. Over time there has been increasing interest in broader measures of diet. Intake of different foods and adherence to different food patterns have been identified as beneficial or non-beneficial for mental health. Studies in adolescence have shown that fruit and vegetables may protect against internalizing and externalizing behaviors [9,10], with authors suggesting mechanisms through certain nutrients like folate, minerals and antioxidants modulating neurotransmitter synthesis or defending against oxidative stress and inflammation. Others demonstrate relations between unhealthy dietary factors, like sugar-sweetened beverages and poor mental health [11]. A systematic review by Nazarian found a decrease in depressive symptoms with a more diverse diet [7].

In a recent systematic review on dietary patterns and depression in the young population, only one study of the 21 presented data from children 5 years or younger [12]. This study was from our group and showed an inverse association between a New Nordic Diet score at ages 3 and 7 years and depression at 8 years [13]. A systematic review by Orlando et al. included two studies with children as young as 4 among the twenty-six included studies [4]. The first study of younger children was from the ALSPAC cohort and showed a link between high intake of junk food at age 4.5 years and increased hyperactivity at 7 years [14]. The second study, which was conducted among 4–12-year-olds from Victoria, Australia, checked the hypothesis that child prosocial behaviors would be positively associated with F&V consumption, and it was supported for both boys and girls. However, the hypothesis that child behavior problems would be associated with low F&V consumption was generally supported for girls but less so for boys, and only with respect to their fruit intake [15]. Campisi et al. reported from the CHILD study in 2026, showing that greater adherence among 5-year-olds to the prudent pattern was associated with fewer internalizing and externalizing problems [16]. Apart from these studies, whereof most are quite old, associations between diet and mental health are typically explored among adults and adolescents and there is a lack of knowledge about the associations between diet and mental health in pre-school children [17]. In addition, we lack studies that have extensive measurements of socio-economic background and maternal mental health, which are likely to confound associations between diet quality and mental health [18,19].

The Early Food for Future Health project was a randomized controlled trial starting at age 6 months and targeting the weaning period and introduction of complementary feeding. Parents of infants in the intervention group were exposed to a digital intervention that ended at child age one year. The included mother–child dyads were followed with questionnaires until age 4 years. This unique data set at 4 years includes dietary information, information on child mental health and thorough data on socio-economic background. The aim of the current paper is to explore the association between different measures of diet quality and child mental health at the age of 4 years.

## 2. Materials and Methods

### 2.1. Study Design

This study was a secondary analysis using cross-sectional data from the randomized controlled trial Early Food for Future Health (EFFH). The primary aim of the original study was to evaluate the effectiveness of a digital dietary intervention targeting the complementary feeding of infants aged 6–12 months, aiming to improve diet quality. Data were collected at 5, 12, 24 and 48 months postpartum. The current study presents cross-sectional dietary and mental health data collected when the included children were 4 years old.

### 2.2. Recruitment and Participants

Parents of infants aged 3–5 months and born after gestational week 38, who were literate in Norwegian and responsible for providing food to their children, were eligible for the study. They were recruited through social media and primary health care centers in Norway [20]. A total of 960 women were recruited, and 715 completed the baseline questionnaire and were randomized into an intervention or control group, respectively. At age 48 months, 444 participants responded to the follow-up questionnaire. All data was collected from web-based, self-administered questionnaires. In the current study we present data from the participants who had filled in dietary data to be used in this study, totaling 363. Characteristics of the participants are provided in Table 1.

### 2.3. Outcome Measures

#### 2.3.1. Child Emotional and Behavioral Problems

The child’s emotional, social and behavioral problems were assessed with the Child Behavioral Checklist (CBCL) [21]. The original CBCL for ages 1½ - 5 consists of 99 problem-items rated on a 3-point scale (0 = not true, 1 = somewhat or sometimes true, 2 = very true or often true). The CBCL1½ - 5 includes seven syndrome scales: Emotional Reactive, Anxious/depressed, Somatic Complaints, Withdrawn, Sleep problems, Attention problems, and Aggressive behavior. In addition, the CBCL 1½ - 5 can be scored into two broader groups of symptoms: internalizing behavior problems (Emotional Reactive, Anxious/depressed, Somatic Complaints, Withdrawn) and externalizing behavior problems (Attention problems and Aggressive behavior). The CBCL is reported to have good reliability and validity [22]. In the current study, an abbreviated version of the full CBCL was used, including 27 items: 16 items addressing internalizing symptoms, and 11 items addressing externalizing symptoms. The abbreviated version of the CBCL was developed for the Norwegian Mother, Father and Child Cohort study (MoBa) [23] and has been used in several previous studies [24]. The MoBa abbreviated externalizing scale is previously found to be representative of the full externalizing CBCL scale [25]. In the present study, parents completed the Child Behavior Checklist (CBCL) at child age four years, from which we summed a total symptom-score for internalizing and externalizing behaviors, respectively.

#### 2.3.2. Dietary Frequency of Consumption

A food frequency questionnaire was developed for this study, compiled and adapted from questionnaires used in the large population-based MoBa study [26] and a national Norwegian dietary survey [27].

Diet was assessed with the following questions: How often does your child drink/eat the following beverages/foods nowadays? The question was asked to capture the frequency of consumption, i.e., how often the child eat or drink a certain food item and not the amount eaten. No explanation or description regarding portion size or quantity was given to the parents. For food items, the response options were never/not tried, less than once per week, 1–2 times/week, 3–4 times/week, 5–6 times/week, once a day, twice a day, 3 times/day, or 4 times per day or more. The alternatives were recoded into a times-per-day score, where never/not tried was recoded into 0 times/day, less than once per week into 0.1 times/day, 1–2 times/week into 0.2 times/day, 3–4 times/week into 0.5 times/day, 5–6 times/week into 0.8 times/day and 4 times per day or more into 5 times/day. Response options for beverages differed slightly from food items, with the options never/seldom, 1–3 times/week, 4–6 times/week, once a day, twice a day, 3 times/day, 4 times/day, or 5 times per day or more. The alternatives were recoded into a times-per-day score, where never/seldom was recoded into 0 times/day, 1–3 times/week into 0.3 times/day, 4–6 times/week into 0.7 times/day, and 5 times per day or more into 6 times/day. Five food scores were computed. The vegetable score was summed from reported frequency of consumption of potatoes, carrots, rutabaga, sweet potatoes, cauliflower, broccoli, green salad, spinach, cucumber, tomatoes, corn, peppers, peas/beans, frozen vegetable mix and salad of mixed raw vegetables (15 items). The fruit score was summed from reported frequency of consumption of orange/clementines, bananas, apples, pears, plums, grapes, kiwi, melon, mango and berries—all types (10 items). The sweet/salty snack score was summed from reported frequency of consumption of cake/cookies, dessert/ice cream, chocolate, sweets and potato chips (5 items), in this paper also referred to as non-core foods. The soft drink score was summed from reported frequency of consumption of sugar-sweetened and artificially sweetened lemonades and soft drinks (4 items). The soft drink score was highly skewed to the left due to a large proportion of the sample having zero frequency of consumption, and it did not meet the assumptions for modeling in linear regression. We therefore recoded the continuous variable into a dichotomized variable by denoting frequency of consumption of soft drinks either never/seldom or 1–3 times/week or more, and applied this variable as exposure in the respective regression models.

### 2.4. Covariates/Potential Confounders

Participating mothers responded to demographic questions related to age, marital status, level of education, financial challenges, and maternal mental health. Maternal marital status was assessed by “What is your marital status?”, with response options recoded to Married/cohabitant or living alone. Maternal education was assessed by asking “What is your highest completed education?”, with seven categories. These were recoded into high or low education (college/university vs. no college/university education). Financial difficulties were assessed with two questions: (1) Do you have the ability to pay an unforeseen expense of 3000 NOK, with response alternatives: ‘yes’, ‘no’ and ‘maybe’. These were recoded into ‘yes’ and ‘no/maybe’. (2) Have you experienced difficulties paying your rent, food or transportation costs during the last 6 months? Possible responses were ‘sometimes’, ‘often’ or ‘never’, subsequently recoded into ‘difficulties’ and ‘no difficulties’. 

The short version (SCL 8) of the Hopkins Symptoms Checklist (SCL 90) was used to assess maternal symptoms of depression and anxiety [28]. This is a well-established psychometric instrument and has previously been used in the MoBa study [29]. Symptoms are assessed by “Have you been bothered by any of the following during the last two weeks?”, with eight symptoms listed. The instrument has four response categories rated from 1 to 4, with higher scores reflecting more severe symptoms. The total score (SCL) was computed by adding the eight item scores and dividing the sum-score by the number of items. Cronbach‘s alpha for this scale in our sample was 0.85, suggesting good internal consistency.

The choice of variables to be included in the multivariable regressions models were based on the literature and previous knowledge of variables that were associated with child diet and causally related to child mental health.

### 2.5. Statistical Approach

All data were analyzed by using IBM SPSS Statistics version 30.0 (IBM Corp., Somers; NY, USA). We used descriptive statistics to present demographic and dietary information about the sample. Continuous variables are presented with mean and standard deviation (SD) regardless of distribution, and frequencies with number (n) and proportion (%). Individual food variables are often not normally distributed, but four of the food scores attained a normal distribution. The soft drink score was highly skewed to the left due to a large proportion reporting frequency of consumption of soft drinks as seldom or never. We therefore chose to recode the continuous soft drink variable into a dichotomized variable with never/seldom = 0 and 1–3 times/week or more = 1.

Associations between the respective summed food scores and internalizing and externalizing behaviors were assed in crude and adjusted linear regression models. We adjusted for randomization status, maternal education level (university education yes/no), ability to handle an immediate expense of 3000 NOK (yes/no), and struggle to handle current expenses (yes/no). In a third model we additionally adjusted for a measure of maternal mental health (SCL-8). Adjustments were made in line with the literature. We checked for violation of assumptions with plots of standardized residuals and Q–Q plots. Assumptions were reasonably met in all statistical models.

To identify potential differences in the associations according to child sex or whether the child had been exposed to the dietary intervention between 6 and 12 months of age or not, we reran the models stratified by randomization status and according to child sex.

## 3. Results

### 3.1. Main Results

A total of 363 children provided dietary data and were included in the analyses. Characteristics of the participating children and their mothers are provided in Table 1. The data set comprised 51.5% girls, and the mean total symptom-score on internalizing behavior was 21.1 and for externalizing behavior it was 16.6. Mean maternal age was 35.8 years, 96% were married/cohabitant, and 87% had completed higher education. In total, 6–12% reported having financial difficulties. Mean maternal symptom-score on the depression and anxiety scale was 1.3.

Mean frequency of consumption of vegetables was 3.7 times/day, fruit and berries 2.7, F&V combined 6.3, sweet/salty snacks 0.7, and any type of sugar-sweetened or artificially sweetened soft drinks 0.4 times/day. Associations between food scores and internalizing behavior are presented in Table 2. The total vegetable score was inversely associated with internalizing behavior in crude and adjusted models. In the model adjusted for maternal education and financial difficulties, a one-unit per day increase in vegetables was associated with a 0.28 points lower symptom-score (β −0.282, 95% CI: −0.481, −0.084). When additionally adjusted for maternal symptoms of depression and anxiety (SCL-8), the association slightly attenuated but was still significant (β −0.254, 95% CI: −0.451, −0.058). The total fruit score (β −0.315, 95% CI: −0.623, −0.007) and the total fruit and vegetable score (β −0.207, 95% CI: −0.351, −0.063) were also significantly and inversely associated with internalizing behavior. The sweet/salty snack score was positively associated and soft drink score inversely with internalizing behavior, but none of these associations were significant (Table 2).

Associations between the food scores and externalizing behavior are presented in Table 3. The vegetable score (β −0.281, 95% CI: −0.481, −0.081) and the total fruit and vegetable score (β −0.188, 95% CI: −0.336, −0.041) were both inversely associated with externalizing behavior in fully adjusted models. The sweet/salty snack score was positively associated with externalizing behavior in crude and adjusted models. In the fully adjusted model, a one-unit per day increase in the sweet/salty snack score was associated with a 1.8 points higher symptom-score (β 1.807, 95% CI: 0.276, 3.337). The total soft drink score was positively associated with externalizing behavior and the fruit score inversely associated, however neither of them were significant.

### 3.2. Sub-Group Analyses

Girls had slightly higher frequency of consumption for fruit and vegetables than boys, but frequency of consumption of sweet/salty snacks and soft drinks did not differ. Associations between the food scores and internalizing behaviors according to child sex are presented in Table 4. The inverse associations found for vegetables and fruit and vegetables combined were significant for boys only (fully adjusted model for vegetables: β −0.306, 95% CI: −0.595, −0.016; fruit and vegetables combined: β −0.225, 95% CI: −0.483, −0.027). Even though not significant, associations were in the same direction and of comparable effect sizes for girls.

Associations between the food scores and externalizing behaviors according to child sex are presented in Table 5. The inverse associations found for vegetables and fruit and vegetables combined were also present for externalizing behavior; however, they were only significant for boys (fully adjusted model for vegetables: β −0.351, 95% CI: −0.663, −0.038; fruit and vegetables combined: β −0.272, 95% CI: −0.518, −0.025). Even though not significant, findings were generally similar and of a comparable effect size for girls.

To exclude the possibility that factors associated with having been exposed to the digital intervention from 6 to 12 months of age might influence our findings, we reran all models confined to the control group. All findings were similar, with no indication of differing associations among intervention participants and controls.

## 4. Discussion

This study is one of few to report on associations between young children’s diet and mental health, as studies are typically performed on older children, adolescents, and adults. We found that frequency of consumption of vegetables and total frequency of consumption of fruit and vegetables in 4-year-old children were inversely associated with internalizing behavior independently of maternal education, measures of financial difficulties and maternal symptoms of depression and anxiety. Further, in the same group, we found that frequency of consumption of non-core foods was positively associated with externalizing behavior, and that frequency of consumption of vegetables was inversely associated; additionally, this was independent of measures of socio-economic position and maternal mental health. Early childhood is an important phase of growth, cognitive and motoric development and a phase that lays foundations for current and future health. Having high intake of fruit and vegetables and limited intake of non-core foods are general dietary advice for both young and adult populations. Our study adds evidence that following this advice may also be associated with better mental health.

Our study, in four-year-olds, shows that having a higher frequency of consumption of fruit and vegetables is inversely associated with internalizing behavior (symptoms of depression, anxiety and emotional problems). This is in line with what is reported among adolescents and results from the MoBa cohort in young children [30]. It is also somewhat in line with Campisi et al., whose findings suggest an inverse relationship between a prudent dietary pattern (high in vegetables, fruits, legumes, eggs and fish) and internalizing problems [16]. In contrast with several other papers on adolescents, we did not find associations between non-core foods and internalizing behavior [4].

There are components in fruit and vegetables that could protect against depressive symptoms by reducing oxidative stress, like vitamins C and E, beta-carotene and folic acid. Neuronal cells in the brain are susceptible to oxidative stress, which may cause neuronal damage and favor occurrence of depression [4]. Vegetables are typically eaten during shared meal settings, like dinners [31]. Shared meals may be positive environments with interactions between parents and children, which may also favor mental health [32]. The observed association between vegetable and fruit frequency of consumption and internalizing behavior might also be an expression of reverse causation, meaning that children with symptoms of depression and emotional problems may be more reluctant to eat certain foods or have more neophobic tendencies towards vegetables or fruits [33] than those without these problems.

We also found that frequency of consumption of fruit and vegetables was inversely associated externalizing behavior and non-core foods was positively associated. Campisi et al. found an inverse association between prudent diet and externalizing behavior, but no association with Western diet, which includes (but is not limited to) the components of the sweet/salty snack score in our study. There are few comparable studies with our age group regarding externalizing behavior. Two studies have found that unhealthy dietary patterns in pregnancy and early infancy are related to later child externalizing behavior [30,34]. Others have reported that children scoring high on externalizing behavior early in life have higher intake of unhealthy food later. The mechanism for why unhealthy foods may lead to externalizing behavior can be that sugars [35] and high-fat diets [36], which independently of nutrient deficiencies, reduce the plasticity in areas of the brain highly relevant for mental health. Taking the possibility of reverse causation into account, one could speculate whether those with externalizing behaviors are more easily given unhealthy foods to be calmed down [37].

The effect estimates related to aspects of non-core foods were larger than for aspects of a healthy diet. Jacka et al. [30] found similar findings in the MoBa study and suggest that the relative contribution from healthy and unhealthy diet to the variance in internalizing and externalizing shows that these patterns are not just the opposite of each other, but that one can be high or low in both healthy and unhealthy dietary dimensions. The pathophysiology for the respective associations may differ, as described above.

Mental health problems are one of Western societies’ increasing challenges, with societal impacts such as educational drop outs, low work capacity, family disruption, loneliness, crime and health challenges, and struggles for each individual [3]. Regardless of numerous policies aiming to reduce such disorders, there has not been much change over the years [3]. Depression and anxiety established early may be difficult to treat later, so providing research from early ages on protective factors and ways to prevent mental health challenges is therefore of the utmost importance. Currently, adherence to dietary guidelines is low [38,39], so finding ways to support the young population by targeting the structures and systems that children are a part of, like kindergarten care and primary health care, in addition to supporting parents in dietary care for their children should be a public health priority.

Strengths and limitations: To measure children’s internalizing and externalizing symptoms, we used validated and well-known instruments, which is a strength of this study. This also holds for the maternal mental health instruments. We have conducted several sub-analyses to get a better understanding of the results. The analyses differentiating on sex showed significant findings in boys only; however the estimates and directions of the associations were similar for both boys and girls. Another strength is the ability to adjust for relevant confounders, especially maternal symptoms of anxiety and depression and the socio-economic variables which cover three important elements of socio-economic position, including education and financial factors. However, even though we adjusted for several variables, we cannot exclude the possibility of residual or unmeasured confounding. It should be noted that the sample is not representative of the general population given the high education level and underrepresentation of low-socio-economic-position groups. A broader representation might entail a wider variety in dietary exposures and potentially reveal even stronger associations with internalizing and externalizing behaviors. Still, it is interesting and of merit that we find these clear associations in a highly educated group.

There are several limitations to this study. First, the data are cross-sectional, which prohibits causal inference. In addition, with this design we cannot say whether diet affects these aspects of mental health or vice versa. If reverse causation explains the observed associations between diet and child internalizing and externalizing behaviors, our findings represent a clear indication that these behaviors are associated with unhealthy dietary aspects that should be acknowledged and highlighted in the dietary care of those with behavioral challenges. Second, data were self-reported, which increases the risk of misreporting and limits the reliability of the conclusions. This holds both for the dietary data and the reporting of maternal and child mental health data. Often, when using food frequency questionnaires people tend to overestimate healthy food items and underreport those perceived as unhealthy [40]. In addition, child diet is difficult to assess, as the care giver might not know all the child eats. Third, the data are not necessarily representative of the diet among 4-year-olds in Norway. Most of the participants were highly educated, which is different from the general parental population in Norway, meaning that there is an underrepresentation from low-socio-economic-position groups. Fourth, we only assessed the frequency of consumption in this study, and there was no assessment of the quantity of each food item. This means that we cannot say how much the children ate of each food item, only how often, which is a limitation and makes our data less precise. We used this approach to reduce participant burden and increase number of participants responding.

## 5. Conclusions

As one of few reporting on young children’s diet and its association with mental health, we found, in a Norwegian study of 4-year-olds, a clear inverse association between child frequency of consumption of fruit and vegetables and internalizing and externalizing behaviors. In addition, we report a relatively strong positive association between sweet/salty snacks score and externalizing behavior. All associations were adjusted for known confounders like maternal mental health and socio-economic background. Mental health problems are costly for the individual and society, and costly to treat. Securing a varied and healthy diet early in life may be a way to promote child mental health, with potential large returns for society. The field needs to identify ways to improve early life diet, for which targeted intervention studies are needed.

## Figures and Tables

**Table 1:** Characteristics of the participating four-year-old children and their mothers, n_tot_ = 363.

Characteristics	Values	Mean (SD) or n (%) ^1^
Children		
Gender (n = 363)	Boy	176 (48.5%)
Dietary variables ^2^		
Vegetable score (n = 363)	Frequency per day	3.7 (2.1)
Fruit score (n = 360)	Frequency per day	2.7 (1.5)
Fruit and vegetable score (n = 360)	Frequency per day	6.3 (2.9)
Sweet/salty snack score (n = 363)	Frequency per day	0.7 (0.3)
Soft drink score (n = 363)	Frequency per day	0.4 (0.5)
Behavioral and emotional problems measured by the Child Behavior Checklist (CBCL) (n = 363)	Internalizing behavior, symptom-score	21.1 (3.5)
	Externalizing behavior, symptom-score	16.6 (3.5)
Mothers		
Age, n = 335	Years	35.8 (4.2)
Marital status (n = 336)	Married/Cohabitant	322 (96%)
	Living alone	14 (4%)
Education (n = 336)	College/university	293 (87%)
	Not college/university	43 (13%)
Financial difficulties (n = 336)	Difficulties handling an unexpected expense of 3000 NOK	22 (6.5%)
	Difficulties meeting ongoing expenses for food, transportation, rent, and the like	41 (12%)
Maternal symptoms of depression and anxiety measured by SCL8 (n = 333)	Symptom-score	1.3 (0.4)

^1^ Valid percentages for categorical variables, and means with SD for continuous variables. ^2^ Dietary variables: The vegetable score is summed from reported frequency of consumption of potatoes, carrots, rutabaga, sweet potatoes, cauliflower, broccoli, green salad, spinach, cucumber, tomatoes, corn, peppers (paprika), peas/beans, frozen vegetable mix and salad of mixed raw vegetables. The fruit score is summed from reported frequency of consumption of orange/clementines, bananas, apples, pears, plums, grapes, kiwi, melon, mango and berries—all types. The total fruit and vegetable score was summed from the vegetable and fruit scores. The sweet/salty snack score is summed from reported frequency of consumption of cake/cookies, dessert/ice cream, chocolate, sweets and potato chips. The soft drink score is summed from reported frequency of consumption of sugar-sweetened and artificially sweetened lemonades and soft drinks and dichotomized to denote seldom/never or 1–3 times/week or more.

**Table 2:** Crude and multivariable linear associations between aspects of child diet at 4 years of age and internalizing behavior, as measured by the Child Behavior Check List (CBCL). Regression coefficient β with 95% CI.

Food Groups ^1^	Frequency/Day		Crude Model n = 363			Adjusted for Socio-economic Position and Randomization ^2^ n = 333			Additional Adjustment for Maternal Mental Health SLC-8 n = 333		
	Mean	SD	β	95% CI	*p*-Value	β	95% CI	*p*-Value	β	95% CI	*p*-Value
Vegetables	3.65	2.07	**−0.292**	**−0.486**, **−0.098**	**0.003**	**−0.282**	**−0.481**, **−0.084**	**0.005**	**−0.254**	**−0.451**, **−0.058**	**0.011**
Fruit and berries	2.67	1.45	−0.275	−0.575, 0.026	0.073	**−0.351**	**−0.666**, **−0.036**	**0.029**	**−0.315**	**−0.623**, **−0.007**	**0.045**
Fruit and Vegetables	6.32	2.92	**−0.225**	**−0.367**, **−0.083**	**0.002**	**−0.232**	**−0.378**, **−0.086**	**0.002**	**−0.207**	**−0.351**, **−0.063**	**0.005**
Sweet/salty snacks	0.74	0.25	0.906	−0.528, 2.340	0.215	1.195	−0.337, 2.732	0.126	1.050	−0.451, 2.551	0.170
Soft drinks	0.40	0.45	−0.169	−0.964, 0.624	0.676	−0.255	−1.076, 0.566	0.541	−0.316	−1.118, 0.486	0.438

^1^ The vegetable score was summed from reported frequency of consumption of 15 vegetables. The fruit score was summed from reported frequency of consumption of ten fruit and berry items. The fruit and vegetable score was summed from the respective summed scores. The sweet/salty snack score was summed from reported frequency of consumption of cake/cookies, dessert/ice cream, chocolate, sweets and potato chips. The soft drink score was summed from reported frequency of consumption of sugar-sweetened and artificially sweetened lemonades and soft drinks and subsequently recoded into a dichotomized variable denoting frequency of consumption of soft drinks seldom/never (=0) vs. 1–3 times/week or more (=1). ^2^ Associations were adjusted for maternal education, financial difficulties, and randomization status. Additional adjustment for maternal mental health measured by SLC-8 is in the right column. Significant associations are indicated by bold text. N = 3 participants had missing values for all fruit and berry items in the FFQ and are not included in the respective models. We excluded one extreme outlier in the vegetable score and two extreme outliers in the fruit score.

**Table 3:** Crude and multivariable linear associations between aspects of child diet at 4 years and externalizing behavior, as measured by the Child Behavior Check List (CBCL). Regression coefficient β with 95% CI.

Food Groups ^1^	Frequency/Day		Crude Model n = 363			Adjusted for Socio-economic Position and Randomization ^2^ n = 333			Additional Adjustment for Maternal Mental Health SLC-8 n = 333		
	Mean	SD	β	95% CI	*p*-Value	β	95% CI	*p*-Value	β	95% CI	*p*-Value
Vegetable score	3.65	2.07	**−0.251**	**−0.447**, **0.055**	**0.012**	**−0.284**	**−0.485**, **−0.083**	**0.006**	**−0.281**	**−0.481**, **−0.081**	**0.006**
Fruit and berries score	2.67	1.45	−0.186	−0.491, 0.118	0.229	−0.230	−0.551, 0.091	0.159	−0.209	−0.526, 0.107	0.195
Fruit and Vegetables	6.32	2.92	**−0.174**	**−0.318**, **−0.030**	**0.018**	**−0.197**	**−0.346**, **−0.049**	**0.009**	**−0.188**	**−0.336**, **−0.041**	**0.012**
Sweet/salty snack score	0.74	0.25	**1.832**	**0.390**, **3.273**	**0.013**	**1.956**	**0.407**, **3.505**	**0.013**	**1.807**	**0.276**, **3.337**	**0.021**
Soft drinks score	0.40	0.45	0.455	−0.348, 1.258	0.266	0.250	−0.584, 1.084	0.556	0.234	−0.589, 1.056	0.576

^1^ The vegetable score was summed from reported frequency of consumption of 15 vegetables. The fruit score was summed from reported frequency of consumption of ten fruit and berry items. The fruit and vegetable score was summed from the respective summed scores. The sweet/salty snack score was summed from reported frequency of consumption of cake/cookies, dessert/ice cream, chocolate, sweets and potato chips. The soft drink score was summed from reported frequency of consumption of sugar-sweetened and artificially sweetened lemonades and soft drinks and subsequently recoded into a dichotomized variable denoting frequency of consumption of soft drinks seldom/never (=0) vs. 1–3 times/week or more (=1). ^2^ Associations were adjusted for maternal education, financial difficulties, and randomization status. Additional adjustment for maternal mental health measured by SLC-8 is in the right column. Significant associations are indicated by bold text. N = 3 participants had missing values for all fruits and berries and are not included in the respective models. We excluded one extreme outlier in the vegetable score and two extreme outliers in the fruit score.

**Table 4:** Multivariable linear associations between aspects of child diet at 4 years and internalizing behaviors, as measured by the Child Behavior Check List (CBCL) stratified by child sex. Regression coefficient β with 95% CI.

Food Group ^1^	Girls n = 173			Boys n = 160		
	β ^2^	95% CI	*p*-Value	β ^2^	95% CI	*p*-Value
Vegetables	−0.225	−0.511, 0.061	0.122	**−0.306**	**−0.595**, **−0.016**	**0.039**
Fruit and berries	−0.375	−0.811, 0.061	0.092	−0.269	−0.730, 0.192	0.250
Fruit and Vegetables	−0.198	−0.399, −0.02	0.052	**−0.255**	**−0.483**, **−0.027**	**0.029**
Sweet/salty snacks	1.224	−1.064, 3.513	0.292	0.924	−1.129, 2.978	0.375
Soft drinks (never/seldom vs. 1–3 time/wk or more)	−0.205	−1.361, 0.950	0.762	−0.394	−1.549, 0.762	0.502

^1^ The vegetable score was summed from reported frequency of consumption of 15 vegetables. The fruit score was summed from reported frequency of consumption of ten fruit and berry items. The fruit and vegetable score was summed from the respective summed scores. The sweet/salty snack score was summed from reported frequency of consumption of cake/cookies, dessert/ice cream, chocolate, sweets and potato chips. The soft drink score was summed from reported frequency of consumption of sugar-sweetened and artificially sweetened lemonades and soft drinks and subsequently recoded into a dichotomized variable denoting frequency of consumption of soft drinks seldom/never (=0) vs. 1–3 times/week or more (=1). ^2^ Associations were adjusted for maternal education, financial difficulties, randomization status, and maternal mental health measured by SLC-8. Significant associations are indicated by bold text. N = 3 participants had missing values for all fruits and berries and are not included in the respective models. We excluded one extreme outlier in the vegetable score and two extreme outliers in the fruit score.

**Table 5:** Multivariable linear associations between aspects of child diet at 4 years and externalizing behaviors, as measured by the Child Behavior Check List (CBCL) stratified by child sex. Regression coefficient β with 95% CI.

Food Groups ^1^	Girls n = 173			Boys n = 160		
	β ^2^	95% CI	*p*-Value	β ^2^	95% CI	*p*-Value
Vegetable score	−0.208	−0.478, 0.062	0.130	**−0.351**	**−0.663**, **−0.038**	**0.028**
Fruit and berries score	−0.105	−0.522, 0.312	0.621	−0.340	−0.840, 0.160	0.181
Fruit and Vegetables combined	−0.126	−0.317, 0.065	0.194	**−0.272**	**−0.518**, **−0.025**	**0.031**
Sweet/salty snack score	1.850	−0.307, 4.008	0.092	1.537	−0.692, 3.766	0.175
Soft drinks (never/seldom vs. 1–3 time/wk or more)	0.439	−0.654, 1.532	0.429	−0.109	−1.370, 1.152	0.865

^1^ The vegetable score was summed from reported frequency of consumption of 15 vegetables. The fruit score was summed from reported frequency of consumption of ten fruit and berry items. The fruit and vegetable score was summed from the respective summed scores. The sweet/salty snack score was summed from reported frequency of consumption of cake/cookies, dessert/ice cream, chocolate, sweets and potato chips. The soft drink score was summed from reported frequency of consumption of sugar-sweetened and artificially sweetened lemonades and soft drinks and subsequently recoded into a dichotomized variable denoting frequency of consumption of soft drinks seldom/never (=0) vs. 1–3 times/week or more (=1). ^2^ Associations were adjusted for maternal education, financial difficulties, randomization status, and maternal mental health measured by SLC-8. Significant associations are indicated by bold text. N = 3 participants had missing values for all fruits and berries and are not included in the respective models. We excluded one extreme outlier in the vegetable score and two extreme outliers in the fruit score.

## Data Availability

Data will be available on DATAVERSE from the University of Agder.

## Abbreviations

The following abbreviations are used in this manuscript:

MoBa	The Norwegian Mother, Father and Child cohort
CBCL	Child Behavioral Checklist
SCL	Hopkins Symptoms Checklist

## References

[1] Gillies N.A., Lovell A.L., Waldie K.E., Wall C.R. (2025). The effect of fruits and vegetables on children’s mental and cognitive health: A systematic review of intervention studies and perspective for future research. Nutrition.

[2] Vergunst F., Commisso M., Geoffroy M.-C., Temcheff C., Poirier M., Park J., Vitaro F., Tremblay R., Côté S., Orri M. (2023). Association of Childhood Externalizing, Internalizing, and Comorbid Symptoms With Long-term Economic and Social Outcomes. JAMA Netw. Open.

[3] World Health Organization (2025). Child and Youth Mental Health in the WHO European Region: Status and Actions to Strengthen the Quality of Care.

[4] Orlando L., Savel K.A., Madigan S., Colasanto M., Korczak D.J. (2022). Dietary patterns and internalizing symptoms in children and adolescents: A meta-analysis. Aust. N. Z. J. Psychiatry.

[5] Arslan İ.B., Lucassen N., van Lier P.A.C., de Haan A.D., Prinzie P. (2021). Early childhood internalizing problems, externalizing problems and their co-occurrence and (mal)adaptive functioning in emerging adulthood: A 16-year follow-up study. Soc. Psychiatry Psychiatr. Epidemiol..

[6] Bayer J.K., Guyon-Harris K.L., Broadley O., Gill A., Hiscock H., Shaw D.S. (2025). Prevalence and Late-Infancy Predictors of Persistent Externalizing Problems During Early Childhood: Child and Family Factors to Consider for Early Screening. Child Fam. Behav. Ther..

[7] Nazarian A., Shidfar F., Dehnad A., Shahinfar H. (2025). Effect of dietary diversity on depression and depressive symptoms: A systematic review and meta-analysis of cross-sectional studies. BMC Public Health.

[8] Cusick S.E., Georgieff M.K. (2016). The Role of Nutrition in Brain Development: The Golden Opportunity of the “First 1000 Days”. J. Pediatr..

[9] Oddy W.H., Robinson M., Ambrosini G.L., O′Sullivan T.A., de Klerk N.H., Beilin L.J., Silburn S.R., Zubrick S.R., Stanley F.J. (2009). The association between dietary patterns and mental health in early adolescence. Prev. Med..

[10] Huang P., O’Keeffe M., Elia C., Karamanos A., Goff L.M., Maynard M., Cruickshank J.K., Harding S. (2019). Fruit and vegetable consumption and mental health across adolescence: Evidence from a diverse urban British cohort study. Int. J. Behav. Nutr. Phys. Act..

[11] Freije S.L., Senter C.C., Avery A.D., Hawes S.E., Jones-Smith J.C. (2021). Association Between Consumption of Sugar-Sweetened Beverages and 100% Fruit Juice With Poor Mental Health Among US Adults in 11 US States and the District of Columbia. Prev. Chronic Dis..

[12] da Silva L.E.M., Costa P.R.F., Brito Beck da Silva Magalhães K., Cunha C.M., Pinheiro de Oliveira Alves W., Miranda Pereira E., de Santana M.L.P. (2025). Dietary Pattern and Depressive Outcomes in Children and Adolescents: Systematic Review and Meta-analysis of Observational Studies. Nutr. Rev..

[13] Vejrup K., Hillesund E.R., Agnihotri N., Helle C., Øverby N.C. (2023). Diet in Early Life Is Related to Child Mental Health and Personality at 8 Years: Findings from the Norwegian Mother, Father and Child Cohort Study (MoBa). Nutrients.

[14] Wiles N.J., Northstone K., Emmett P., Lewis G. (2009). ‘Junk food’ diet and childhood behavioural problems: Results from the ALSPAC cohort. Eur. J. Clin. Nutr..

[15] Renzaho A.M., Kumanyika S., Tucker K.L. (2011). Family functioning, parental psychological distress, child behavioural problems, socio-economic disadvantage and fruit and vegetable consumption among 4–12 year-old Victorians, Australia. Health Promot. Int..

[16] Campisi S.C., Chen Z.H., Simons E., Mandhane P., Moraes T.J., Turvey S.E., Subbarao P., Miliku K., Korczak D.J. (2026). Associations Between Dietary Patterns and Mental Health Symptoms in Early Childhood: Findings From the CHILD Cohort Study. J. Dev. Behav. Pediatr..

[17] Sparling T.M., Deeney M., Cheng B., Han X., Lier C., Lin Z., Offner C., Santoso M.V., Pfeiffer E., Emerson J.A. (2022). Systematic evidence and gap map of research linking food security and nutrition to mental health. Nat. Commun..

[18] Khalid S., Williams C.M., Reynolds S.A. (2016). Is there an association between diet and depression in children and adolescents? A systematic review. Br. J. Nutr..

[19] O’Neil A., Quirk S.E., Housden S., Brennan S.L., Williams L.J., Pasco J.A., Berk M., Jacka F.N. (2014). Relationship between diet and mental health in children and adolescents: A systematic review. Am. J. Public. Health.

[20] Helle C., Hillesund E.R., Wills A.K., Øverby N.C. (2019). Evaluation of an eHealth intervention aiming to promote healthy food habits from infancy -the Norwegian randomized controlled trial Early Food for Future Health. Int. J. Behav. Nutr. Phys. Act..

[21] Achenbach T.M., Edelbrock C. (1983). Manual for the Child Behavior Checklist and Revised Child Behavior Profile.

[22] Kristensen S., Henriksen T.B., Bilenberg N. (2010). The Child Behavior Checklist for ages 1.5–5 (CBCL/1½–5): Assessment and analysis of parent-and caregiver-reported problems in a population-based sample of Danish preschool children. Nord. J. Psychiatry.

[23] Nøvik T.S. (1999). Validity of the Child Behaviour Checklist in a Norwegian sample. Eur. Child. Adolesc. Psychiatry.

[24] Oerbeck B., Overgaard K.R., Pripp A.H., Reichborn-Kjennerud T., Aase H., Zeiner P. (2020). Early Predictors of ADHD: Evidence from a Prospective Birth Cohort. J. Atten. Disord..

[25] Zachrisson H.D., Dearing E., Lekhal R., Toppelberg C.O. (2013). Little evidence that time in child care causes externalizing problems during early childhood in Norway. Child. Dev..

[26] Magnus P., Birke C., Vejrup K., Haugan A., Alsaker E., Daltveit A.K., Handal M., Haugen M., Høiseth G., Knudsen G.P. (2016). Cohort Profile Update: The Norwegian Mother and Child Cohort Study (MoBa). Int. J. Epidemiol..

[27] Norwegian Health Directorate (2009). Nationwide Diet Survey Among 12 Months Old Children.

[28] Derogatis L.R., Lipman R.S., Covi L. (1973). SCL-90: An outpatient psychiatric rating scale--preliminary report. Psychopharmacol. Bull..

[29] Tambs K., Røysamb E. (2014). Selection of questions to short-form versions of original psychometric instruments in MoBa. Nor. Epidemiol..

[30] Jacka F.N., Ystrom E., Brantsaeter A.L., Karevold E., Roth C., Haugen M., Meltzer H.M., Schjolberg S., Berk M. (2013). Maternal and early postnatal nutrition and mental health of offspring by age 5 years: A prospective cohort study. J. Am. Acad. Child. Adolesc. Psychiatry.

[31] Hillesund E.R., Sagedal L.R., Bere E., Øverby N.C. (2021). Family meal participation is associated with dietary intake among 12-month-olds in Southern Norway. BMC Pediatr..

[32] Harrison M.E., Norris M.L., Obeid N., Fu M., Weinstangel H., Sampson M. (2015). Systematic review of the effects of family meal frequency on psychosocial outcomes in youth. Can. Fam. Physician.

[33] Wallace G.L., Llewellyn C., Fildes A., Ronald A. (2018). Autism spectrum disorder and food neophobia: Clinical and subclinical links. Am. J. Clin. Nutr..

[34] Steenweg-de Graaff J., Tiemeier H., Steegers-Theunissen R.P., Hofman A., Jaddoe V.W., Verhulst F.C., Roza S.J. (2014). Maternal dietary patterns during pregnancy and child internalising and externalising problems. The Generation R Study. Clin. Nutr..

[35] Xiong J., Wang L., Huang H., Xiong S., Zhang S., Fu Q., Tang R., Zhang Q. (2024). Association of sugar consumption with risk of depression and anxiety: A systematic review and meta-analysis. Front. Nutr..

[36] Adan R.A.H., van der Beek E.M., Buitelaar J.K., Cryan J.F., Hebebrand J., Higgs S., Schellekens H., Dickson S.L. (2019). Nutritional psychiatry: Towards improving mental health by what you eat. Eur. Neuropsychopharmacol..

[37] Warnick J.L., Stromberg S.E., Krietsch K.M., Janicke D.M. (2019). Family functioning mediates the relationship between child behavior problems and parent feeding practices in youth with overweight or obesity. Transl. Behav. Med..

[38] Myhre J.B., Brodin M.M.A., Andersen L.F. (2024). Norkost 4 A National Diet Study.

[39] Warkentin S., Stratakis N., Fabbri L., Wright J., Yang T.C., Bryant M., Heude B., Slama R., Montazeri P., Vafeiadi M. (2025). Dietary patterns among European children and their association with adiposity-related outcomes: A multi-country study. Int. J. Obes..

[40] Calvert C., Cade J., Barrett J.H., Woodhouse A. (1997). Using cross-check questions to address the problem of mis-reporting of specific food groups on Food Frequency Questionnaires. Eur. J. Clin. Nutr..

